# Differential heme release from various hemoglobin redox states and the upregulation of cellular heme oxygenase‐1

**DOI:** 10.1002/2211-5463.12103

**Published:** 2016-08-08

**Authors:** Tigist Kassa, Sirsendu Jana, Fantao Meng, Abdu I. Alayash

**Affiliations:** ^1^ Laboratory of Biochemistry and Vascular BiologyCenter for Biologics Evaluation and ResearchFood and Drug AdministrationSilver SpringMDUSA

**Keywords:** ferric, ferryl hemoglobin, heme, hemoglobin

## Abstract

Despite advances in our understanding of the oxidative pathways mediated by free hemoglobin (Hb), the precise contribution of its highly reactive redox forms to tissue and organ toxicities remains ambiguous. Heme, a key degradation byproduct of Hb oxidation, has recently been recognized as a damage‐associated molecular pattern (DAMP) molecule, able to trigger inflammatory responses. Equally damaging is the interaction of the highly redox active forms of Hb with other biological molecules. We determined the kinetics of heme loss from individual Hb redox states—ferrous (Fe^2+^), ferric (Fe^3+^), and ferryl (Fe^4+^)—using two different heme receptor proteins: hemopexin (Hxp), a naturally occurring heme scavenger in plasma, and a double mutant (H64Y/V86F), apomyoglobin (ApoMb), which avidly binds heme released from Hb. We show for the first time that ferric Hb (Fe^3+^) loses heme at rates substantially higher than that of ferryl Hb (Fe^4+^). This was also supported by a higher expression of heme oxygenase‐1 (HO‐1) when ferric Hb was added to cultured lung alveolar epithelial cells (E10). The reported cytotoxicity of Hb may therefore be attributed to a combination of accelerated heme loss from the ferric form and protein radical formation associated with ferryl Hb. Targeted therapeutic interventions can therefore be designed to curb specific oxidative pathways of Hb in hemolytic anemias and when Hb is used as an oxygen‐carrying therapeutic.

**Abbreviations**
ApoMb: Apomyoglobin, heme free myoglobin
H_2_O_2_: Hydrogen peroxide
HBOCs: Hb‐based oxygen carriers
Hpx: Hemopexin

Hemoglobin (Hb) is normally sequestered within circulating red blood cells (RBCs) as an oxygen carrier for maintaining adequate supplies of oxygen to tissues. To maintain maximum oxygen‐carrying capacity, Hb must be kept under reducing conditions (i.e., in the ferrous (Fe^2+^) formed by an efficient enzymatic machinery, that includes enzymes such catalase, superoxide dismutase, glutathione, and red cells peroxiredoxin‐2 (PRDX2). In a cell‐free environment (during hemolysis due to disease or RBC lesions associated with the aging of RBCs and when used as oxygen‐carrying blood substitutes) Hb has been shown to undergo accelerated oxidation and oxidative changes which can lead ultimately to heme loss to tissues 1.

Hemoglobin undergoes spontaneous oxidation from the ferrous (Fe^2+^) to the ferric (Fe^3+^) states in the presence of air equilibrated aqueous buffer and produces superoxide anions (O_2_·¯) 2. The released O_2_·¯ then undergoes dismutation to H_2_O_2_ and O_2_, and the H_2_O_2_ produced in this step can react with the protein to produce several other species 3. This spontaneous conversion of Hb is broadly classified as autooxidation.

$${\text{HbFe}}^{2+}O_{2}\to {\text{HbFe}}^{3+}+{O_{2}}^{\cdot -}\text{(Autooxidation)}$$

In the presence of self‐generated oxidants during the autooxidation process or produced by other cellular sources, a faster and more damaging sequence of events can be initiated that can result in oxidative changes within the protein. Oxidants such as H_2_O_2_ drive a catalytic cycle known as the pseudoperoxidase reactions (1–3 reactions) that includes the formation of a transient oxoferryl Hb, when the reaction starts with ferrous Hb (Reaction 1). The ferryl species autoreduces to ferric iron (Fe^3+^) (Reaction 2) and in the presence of additional H_2_O_2_, ferryl iron (Fe^4+^) is regenerated back in a classic cycle reported for Hb 4. When H_2_O_2_ reacts with the ferric form of Hb, a protein radical is also formed (Reaction 3), and this radical migrates and further damages the protein, including the oxidation of βCys‐93 and dimerization.

$${\text{HbFe}}^{2+}O_{2}+H_{2}O_{2}\to {\text{HbFe}}^{4+}=O+H_{2}O+O_{2}\;\;\left(\text{Reaction}1\right)$$

$$\begin{array}{c} {\text{HbFe}}^{4+}=O+e^{-}+2H^{+}\to {\text{HbFe}}^{3+}+H_{2}O\;\;\;\;\text{(Reaction 2)} \end{array}$$

$${\text{HbFe}}^{3+}+H_{2}O_{2}\to^{\cdot +}{\text{HbFe}}^{4+}=O+H_{2}O\;\;\;\text{(Reaction 3)}$$

These reactions were described for normal Hb, variants of Hb from a variety of hemoglobinopathies 5, 6 and for a number of Hb‐based oxygen carriers (HBOCs) 7. The most common features among all of these proteins is the persistent ferryl heme and its radical which may be due to mutation effects and/or chemical modifications in the case of some HBOCs. The longevity of the ferryl heme results in wider distribution of damage and oxidative milieu leading to perpetuating side reactions that ultimately lead to protein destabilization as a consequence of (a) heme attachment to nearby amino acids; (b) irreversible oxidation of amino acids in the oxidation hotspot, particularly the βCys93 side chain; (c) structural instability that leads to heme loss, and finally (d) oxidative damage to other cellular and subcellular entities such as the mitochondria 6.

Hemoglobin toxicity as exemplified in the reactions (1–3) have been investigated in cell culture experiments with conflicting outcomes as to the precise contribution of Hb oxidation intermediates and/or heme release from the protein to overall toxicity. These experiments are complicated by several variables including uncontrollable autooxidation (Fe^2+^ → Fe^3+^), autoreduction (Fe^4+^ → Fe^3+^) among Hb species, and protein unfolding; controlling these variables has therefore become an important element in the validity of these studies.

In a murine sickle cell disease model it was recently shown that heme, released from infused ferric Hb rather than ferrous form triggers TLR4 signaling leading to endothelial cell activation and vaso‐occlusion. Coinfusion of haptoglobin (protein scavenger) and hemopexin (heme scavenger) were effective in reversing these inflammatory responses 8. More recently, microparticles laden with Hb were found to be a source for heme released into the microvasculature 9. Based on these observations, it is therefore not surprising that heme derived from Hb/RBC is now recognized as a damage‐associated molecular pattern molecule (DAMP) driving inflammation 8. Understanding the processes in Hb's denaturation has taken on greater importance as a result of attempts to produce blood substitutes based on extracellular Hb. Clinical studies with some of these blood substitutes have revealed symptoms that may be due to accelerated autooxidation and heme dissociation 10.

We have therefore set out to determine the rates of heme loss from these key redox forms of Hb. We have used two different heme receptors to monitor the kinetics of heme transfer from ferric and ferryl forms of Hb. These are hemopexin (Hxp), a naturally occurring heme scavenger in plasma and a double mutant (H64Y/V86F) apomyoglobin (ApoMb), which avidly binds heme released from Hb. The affinity constant value for the binding of heme to the single binding center of hemopexin molecule was estimated to be 1.9 × 10^14^ m ^−1^ 11, whereas the affinity of apomyoglobin for heme was reported to be 8 × 10^13^ m ^−1^ 12. In spite of these differences among these two heme receptors, we consistently showed in this report that ferric looses heme more readily than the ferryl form of Hb. Therefore, the toxicity associated with ferric Hb resides in its susceptibility to loose heme more readily than its higher oxidation state counterpart, the ferryl heme. The toxicity of the ferryl on the other hand lies within its higher redox potential and reactivity toward itself and other biological molecules as we have recently shown 6, 13.

## Experimental procedures

### Preparations of hemoglobin redox forms

Normal blood was obtained by patient's consent from the Division of Transfusion Medicine, National Institutes of Health (NIH), Bethesda, MD, USA. Hemolysate from normal human blood was purified from the human erythrocytes by DEAE chromatography 14. Catalase activity assays were performed on all Hb samples to confirm the complete removal of catalase 15. Catalase and K_3_Fe(CN)_6_ were purchased from Sigma Aldrich (St. Louise, MO, USA). Hemopexin (Hpx) was purchased from Athens Research and Technology (Athens, GA, USA). Hydrogen peroxide (H_2_O_2_) (30% w/w) was purchased from Sigma. Dilute solutions of H_2_O_2_ were prepared fresh daily from a stock by making appropriate dilution in deionized water and kept on ice. The concentration of H_2_O_2_ was determined spectrophotometrically at 240 nm using a molar extinction coefficient of 43.6 m ^−1^·cm^−1^ 16. Double‐mutant myoglobin (Mb) (H64Y/V68F) was a gift from J.S. Olson, Rice University.

Highly purified and well characterized redox forms of human Hb were used in this study and the extinction coefficients of each Hb species (ferrous/oxy, ferric/met and ferryl) were determined. Ferrous Hb was oxidized to ferric (met) Hb by adding 1.5 equivalents of potassium ferricyanide at pH 7.0 at and room temperature. Samples were desalted using a Sephadex G‐25 equilibrated in u50 mm of potassium phosphate buffer pH 7.4 17. Ferryl Hb was prepared by incubating 60 μm of metHb with 600 μm of H_2_O_2_ for 1 min at room temperature followed by addition of 200 units·mL^−1^ catalase to remove excess H_2_O_2_. Spectral identification and verification of Hb species were carried out as previously described 13. The extinction coefficients used to determine concentrations of different Hb redox solutions in 50 mm phosphate buffer pH 7.4 were: 141.2 mm ^−1^·cm^−1^ at 415 nm, 15.5 mm ^−1^·cm^−1^ at 540 nm, and 16.6 mm ^−1^·cm^−1^ at 576 nm for HbO_2_ in heme equivalents; 167.0 mm ^−1^·cm^−1^ at 406 nm, 7.05 mm ^−1^·cm^−1^ at 540 nm, and 3.78 mm ^−1^·cm^−1^ at 630 nm for ferric Hb in heme equivalents; 113.4 mm ^−1^·cm^−1^ at 418 nm, 11.1 mm ^−1^·cm^−1^ at 544 nm, and 9.56 mm ^−1^·cm^−1^ at 576 nm for ferryl Hb in heme equivalents (F. Meng and A. Alayash, unpublished data). These values are close to those reported earlier 18. Spectrophotometric measurements were made with an 8453 Agilent Spectrophotometer, and spectra between 350 and 700 nm were recorded every 2 min for 16 h at 37 °C.

### Kinetics of heme loss from hemoglobins

To assess the rate of heme transfer, we measured the absorbance changes with a heme acceptor, either a double‐mutant Mb (H64Y/V86F) or hemopexin (Hpx) were used. His 64(E7) is replaced by Tyr in sperm whale Mb producing a holoprotein with distinct green color due to an intense absorption band at 600 nm. Val 68(E11) was replaced by Phe in the same protein to increase its stability. When this double‐mutant apoglobin is mixed with either metMb or metHb, a colorimetric change occurs (the brown solution turns green); the resulting absorbance changes can be used to measure complete time courses for heme dissociation from either holoMb or holoHb 19.

Spectra between 350 and 700 nm were recorded every 2 min for 16 h at 37 °C using 200 mm potassium phosphate buffer pH 7.0 with 600 mm sucrose to avoid degradation of the mutant Mb. Final concentration of Hb in heme equivalents was 2 μm and the final concentration of 20 μm of H64Y/V86F was used. For heme transfer to Hpx we followed early reported procedures with minor modifications 11, 20. Sodium phosphate buffer (100 mm, pH 7.0) was used for the Hpx experiments. Final concentration of Hb in heme equivalents were 2 μm and the final concentrations of Hpx were 4 μm in a 1 mL total reactions volume. Hb solutions in three different oxidation states were incubated with Hpx at 37 °C for 16 h. Ferryl Hb was prepared fresh for each experiment by adding 10 equivalents of H_2_O_2_ to 60 μm metHb solution at room temperature. Our rate constants for heme loss were measured at low Hb concentrations in order to facilitate heme dissociations from Hb dimers. At these low Hb concentrations, more than 70% of the protein will be in the dimeric form 21.

### Cell culture and hemoglobin treatments

Mouse type I alveolar epithelial cells (E10) were cultured in CMRL‐media supplemented with 10% fetal bovine serum (v/v), 100 units·mL^−1^ of penicillin, and 100 μg·mL^−1^ of streptomycin in a humidified atmosphere containing 5% CO_2_/95% air. The media were changed every 48 h and up to 80–90% confluent culture dishes with cells grown in complete media were used for Hb exposure. The cells were kept in serum‐free media overnight and then were exposed to different Hb redox states for 12 or 24 h.

### Immunocytochemistry of heme oxygenase and confocal microscopy

To assess the HO‐1 expression in Hb‐treated E10 cells using immunocytochemistry, cells were grown in the complete media up to 50% confluence on glass coverslips. Following incubations with various Hb species at equimolar (100 μm) concentration for 12 h, cells were fixed in 4% paraformaldehyde. Immunocytochemical staining was performed with mouse anti‐HO‐1 primary antibody (Abcam, Cambridge, MA, USA) followed by secondary goat anti‐mouse Alexa Fluor 488 conjugated antibody for HO‐1 as described earlier 6. Cellular actin cytoskeleton was counterstained with Phalloidin Alexa‐647 (red) to visualize the cellular morphology. Cells were visualized under a Zeiss LSM710 meta confocal microscope (Zeiss, Thornwood, NY, USA) after mounting with Fluoroshield mounting medium with DAPI (Abcam).

### HO‐1 immunoblotting

Following incubation with different Hb proteins for 24 h, E10 cells were lysed in RIPA Lysis and Extraction Buffer (Thermo Fisher Scientific Inc., Waltham, MA, USA) and cell lysate proteins were separated on 4–12% NuPAGE bis‐tris precast polyacrylamide gels (Thermo Fisher Scientific Inc.) and were then transferred to a nitrocellulose membrane using standard immunoblotting techniques. Blots were then probed with mouse monoclonal anti‐hemoxygenase‐1 primary antibody and with rabbit monoclonal β‐actin antibody (Abcam). The proteins were visualized using an enhanced chemiluminiscense kit (GE Healthcare, Piscataway, NJ, USA) after probing with HRP‐conjugated secondary antibodies. Bands were visualized using a ChemiDoc Touch Imaging System (Bio‐Rad Laboratories, Inc., Hercules, CA, USA) and quantified by software (Bio‐Rad Laboratories, Inc.). The band intensity of HO‐1 was normalized with corresponding β‐actin and values were expressed as relative band intensity.

### Statistical analysis

All values from the densitometric analysis of western blot bands were expressed as mean ± SEM. Statistical analyses were done using Student's *t*‐test, unpaired. A value of *P* < 0.05 was considered significant.

## Results

### Kinetics of heme loss from redox forms of hemoglobin

To assess the rates of heme transfer, we first measured the absorbance changes when the acceptor molecule, ApoMb, binds the heme released from ferrous, ferric and ferryl forms of Hb to yield holoMb. ApoMb exhibits distinct spectral properties as it completely extracts heme from Hb (and Mb) yielding absorbance changes large enough to allow reactions at low heme concentrations 19. These changes include a decrease in intensity, slight red‐shift of the Soret maximum and the appearance of an intense band at 600 nm which gives a green color. The intense absorption at 600 nm is attributed to the direct coordination of the tyrosine phenol side chain to the iron atom 22.

**Figure 1:**
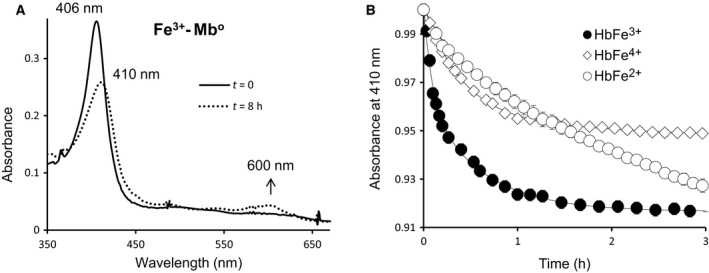
Spectral changes during heme transfer from methemoglobin to the mutant apomyoglobin (H64Y/V86F). (A) Absorbance spectra of metHb (solid line) and H64Y/V86F Mb (dashed line). (B) Time courses for heme dissociation from ferrous, ferric, and ferryl Hb. Reactions were carried out in 200 mm phosphate buffer at pH 7.0 with 600 mm sucrose by mixing 2 µm Hb with 20 µm H64Y/V86F apomyoglobin and time courses were monitored at 410 nm at 37 °C for 16 h.

Figure 1A confirms that heme transfer from Hb to H64Y/V86F to apomyoglobin resulted in a shift in the Soret peak and an increase in absorbance at 600 nm (dashed line). Time courses for the three oxidation states of Hb shown in Fig. 1B were biphasic with fast components representing heme loss from β‐subunits. Both α and β subunits lose their heme at different rates, β being the fastest to lose its heme 23, 24. Estimates of averaged absorbance changes at the Soret peak at 410 nm were normalized and the initial phases of absorbance were fitted to double exponential decay expressions, where *k*_1_ and *k*_2_ are the fast and slow first‐order observed rate constants. Calculated rate constants for the ferrous heme were *k*_1_ = 3.46 h^−1^ and *k*_2_ = 0.20 h^−1^, ferric heme, *k*_1_ = 11.89 h^−1^ and *k*_2_ = 1.69 h^−1^, and for the ferryl heme, *k*_1_ = 2.42 h^−1^ and *k*_2_ = 0.11 h^−1^, respectively (Table 1). These results confirm that ferric Hb loses its heme at rate about 3.4‐fold faster than ferrous and fivefold faster than the ferrylHb.

**Table 1:** First‐order rate constants of heme dissociation from different redox forms of hemoglobin

	Mb^o^ (h^−1^)	Hpx (h^−1^)
HbFe^2+^	*k*_1_ = 3.46 ± 0.12	*k*_1_ = 8.70 ± 0.21
	*k*_2_ = 0.20 ± 0.05	*k*_2_ = 0.55 ± 0.3
HbFe^3+^	*k*_1_ = 11.89 ± 0.16	*k*_1_ = 25.64 ± 0.18
	*k*_2_ = 1.69 ± 0.19	*k*_2_ = 2.3 ± 0.11
HbFe^4+^	*k*_1_ = 2.42 ± 0.07	*k*_1_ = 2.04 ± 0.15
	*k*_2_ = 0.11 ± 0.12	*k*_2_ = 0.15 ± 0.03

**Figure 2:**
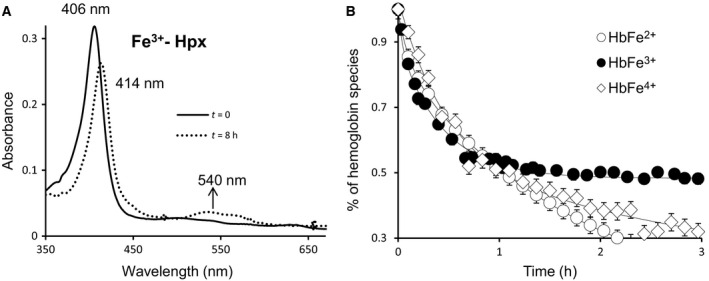
Spectral changes during heme transfer from methemoglobin to hemopexin. (A) Absorbance spectra of metHb (solid line) and Hpx (dashed line). (B) Time courses for heme dissociation from ferrous, ferric, and ferryl Hb. Reactions were carried out in 100 mm sodium acetate buffer at pH 7.0 and by mixing 2 µm Hb with 4 µm Hxp and time courses representing the percentages of remaining ferrous, ferric, and ferryl species in solutions as heme is transferred to Hpx.

To confirm this observation, we measured heme transfer rates from Hb redox forms using Hpx as the acceptor molecule in another sets of experiments. Spectral changes during heme exchange between ferric Hb and Hpx are shown in Fig. 2A. The heme transfer is indicated by the shift in the Soret peak and increase in the absorbance at the visible region 540 nm (Fig. 2A). The shift in Soret peak, approximately 8 nm is in agreement with early reports 11. Time courses for heme loss from Hb redox forms (ferrous, ferric, and ferryl) are shown in Fig. 2B. Rates of heme transfer to Hpx were calculated by plotting the percentages of each redox species (%ferrous, %ferric and %ferryl) in solution as heme is transferred as a function of time to Hpx 20. Rates were calculated by fitting the spectral changes to double exponential decay expressions where *k*_1_ and *k*_2_ are the fast and slow first‐order observed rate constants. Rate constants for ferrous, *k*_1_ = 8.70 h^−1^ and *k*_2_ = 0.55 h^−1^, for ferric, *k*_1_ = 25.64 h^−1^ and *k*_2_ = 2.3 h^−1^, and for ferryl, *k*_1_ = 2.04 h^−1^ and *k*_2_ = 0.15 h^−1^, respectively. These results confirm that ferric Hb loses heme at a rate about 2.9‐fold faster than ferrous and approximately tenfold faster than that of the ferryl. These data also substantiate the data generated by the use of recombinant Mb as the receptor protein and clearly confirm that ferric/metHb loses heme at much higher rates that the ferryl heme.

**Figure 3:**
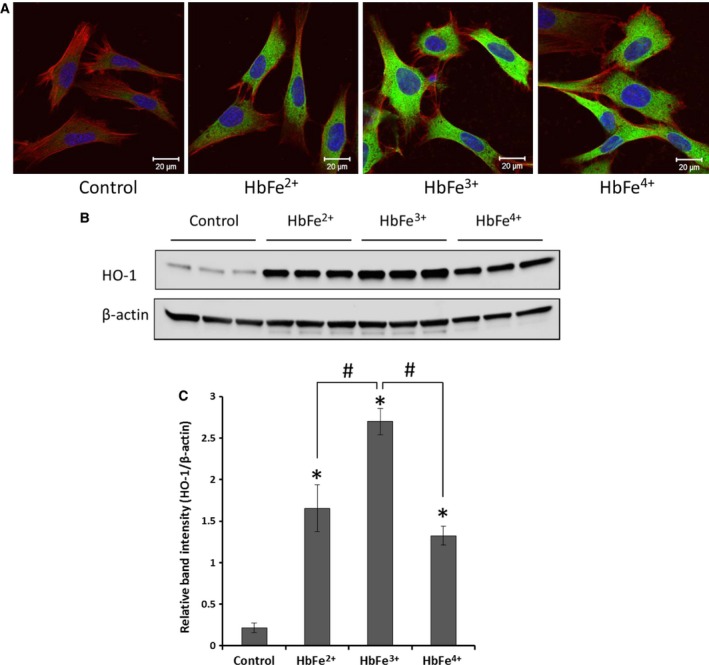
Hemoglobin‐mediated induction of heme oxygenase‐1 (HO‐1) expressions in mouse E10 cells. E10 cells were exposed to either ferrous, ferric, or ferryl Hb at equimolar concentration (100 µm) for 12 h for immunocytochemistry experiment (A) or 24 h for western blotting experiment (B). Laser confocal images showing expression of heme oxygenase‐1 (green, Alexa‐488) in E10 cells (A). E10 cells were also counterstained with Phalloidin Alexa‐647 (red) to show the cellular morphology. Nuclear staining was done with DAPI (blue). Cell lysates were immunoblotted and analyzed for HO‐1 expression (B, upper panel). Equal loading was confirmed by reprobing the blots against β‐actin (B, lower panel). The ratio of average (*n* = 3) band intensity of HO‐1 with corresponding β‐actin was plotted as relative intensity (C), *P < 0.05 vs. control; ^#^ *P* < 0.05 vs. HbFe^3+^, ‘*t*’ test unpaired.

To assess the relative contribution of heme release from various Hb redox proteins on the oxidative defense mechanisms in cultured cells, we monitored the expression of heme‐sensitive heme oxygenase (HO‐1) in mouse E10 cells first by immunocytochemical staining. Mouse E10 cells were exposed to equal concentration (100 μm) of ferrous, ferric, and ferryl Hbs for 12 h and analyzed HO‐1 expression under a laser confocal microscope. Figure 3A shows a representative images indicating profound Hb induced HO‐1 expression (green) over untreated control cells. Both ferric and ferryl species induced more HO‐1 protein expression than the corresponding ferrous species probably through higher heme release. There was slight noticeable differences between ferric and ferryl Hb panels in Fig. 3A. To assess the HO‐1 level more quantitatively, we performed western blotting of cell lysates from E10 cells that had been exposed to equimolar (100 μm) Hb of species for 24 h (Fig. 3B). Densitometric analysis of western blots of cell lysates indicated that untreated control cells did not express any significant HO‐1 protein, whereas (under similar incubation conditions) all three species of Hb caused significant HO‐1 induction in E10 cells (Fig. 3C). However, ferric Hb induced HO‐1 expression was significantly higher than either ferrous or ferryl species.

## Discussion

Classic pseudoperoxidase activities (Reactions 1–3) have been shown to vary among Hbs due in part to conformational and/or mutational variations in these proteins. To protect against Hb oxidative pathways associated with hemolytic anemias (i.e., red cell lesions and when Hb is used as oxygen therapeutic) recent focus has been on controlling the toxicity associated with ferryl/ferryl radical species under conditions, where Hb is free in circulation 7, 25. The slower autoreduction of ferryl to ferric (as we have recently reported) plays a key role in the oxidative toxicity associated with sickle cell Hb at cellular and subcellular levels 6. Slower transition of ferryl back to ferric either through autoreduction itself or through reduction by a cellular component leads to the wider distribution of damage as ferryl Hb persists longer. However when the transfers are rapid, lesser damage may occur to the protein and exogenous species.

Because of the transient nature of the ferryl, the toxicity associated with this species may be limited to its immediate environment and whether or not oxidants are available to fuel Hb pseudoperoxidase cycle. Ferric Hb on the other hand can reach to considerable and measurable levels in circulation with more lasting effects. Oxidized Hbs are generally less stable than the ferrous forms due to unfolding of the proteins and subsequent heme loss and are therefore more amenable for the study of heme release kinetics 26, 27. In deoxyHb, the proximal HisF8‐Fe^2+^ bond is at least 100× stronger than the ferric (HisF8‐Fe^3+^ bond) 28, 29. Our recent study indicated that heme derived from infused ferric Hb in sickle cell animal model can act as a damage‐associated molecular pattern (DAMP) molecule triggering inflammatory responses. Infusion of cyanometHb (in which the iron is blocked) prevented these responses 8.

Several animal studies have reported the accumulation of large quantities of oxidized Hb (ferric) (30–40%) after infusion of ferrous Hb or chemically modified Hb‐based oxygen carries (HBOCs) 30, 31. Recently it was shown that intravascular hemolysis in guinea pigs and beagle dogs (with acute kidney injury) led to intrarenal conversion of ferrous to ferric Hb, accumulation of free heme and Hb‐cross‐linked products, enhanced 4‐hydroxynonenal reactivity in renal tissue, and acute tubule injury. These adverse changes were completely prevented by haptoglobin treatment 32. In a case of compassionate use of one HBOC (in a severely injured Jehovah's Witness patient for whom survival was considered unlikely) the severe anemia and cardiac hypoxia were reversed by slow infusion of Hb and the administration of ascorbate to control ferric Hb accumulation in this patient's circulation 33.

Recent attempts in reversing Hb oxidative toxicity associated with the use of Hb‐based oxygen therapeutics included the introduction of oxidative stable mutation that can eventually be designed into viable oxygen therapeutics. Hb providence (βLys82 → Asp) 34, for example, shows an improved pseudoperoxidase activity which has been proposed as a candidate for such technology 35. Another approach includes the designing of a Hb molecule with tyrosine residues at position 42 in the β subunits as a source of electrons capable of reducing ferryl back to ferric 36.

Using highly purified Hb in the ferrous, ferric and ferryl forms we show for the first time that ferric forms of Hb loses heme at higher rates than ferrous or ferryl. Exposure of E10 cells under our experimental conditions to ferric resulted in a higher expression of HO‐1 when compared to ferrous Hb which may be due to these differences in the rates of heme release from the two proteins. Exposure of these cells to the ferryl on the other hand, elicited a robust increases in HO‐1 expression when compared to ferrous, but these expressions were much lesser in intensity than HO‐1 content induced by the ferric form.

Structural differences among the three oxidation states near the proximal His bond to heme or at the CD corner (near the heme pocket) plays a critical role in determining heme dissociation from Hb and Mb 37. It has been shown that the strength of Fe‐HisF8 bond and water accessibility are key factors governing heme dissociation from these proteins. The faster rate of heme loss from metHb is probably due to structural fluctuations which allow water to enter the proximal pocket and hydrolyze the linkage between globin and heme 27, 38. In the case of the ferryl, which has back bonding to the iron atom via the proximal His is more stable and harder for the heme to dissociate until the protein is unfolded or is reduced back to aquomet forms. The ferryl iron is positioned about 0.3 Å out of the heme plane in the direction of the distal histidine; the proximal histidine moves with it to bring it closer to the heme plane. For ferryl the distance from the iron atom to the center of the ligand electron density is 1.9 Å, which is shorter than the normal Fe‐O distance of 2.2–2.3 Å in most aquo‐metHb and metMb structures. The modeled Fe‐O distance of 1.9 Å is consistent with previously reported ferryl complexes in peroxidases 39. The occupancy of water coordinated to the iron atom of metHb may make it less stable. The persistence in solutions of the ferryl iron on the other hand and its radical protein are, however, more damaging to biological molecules and tissues than the ferric Hb 6. It is therefore important to take these factors in consideration when antioxidant or other interventions are designed against Hb oxidative side reactions and heme loss.

## Author contributions

TK, SJ, and FM performed the experiments and data analysis. All contributed to the writing of the manuscript.
